# Evaluation of the immunogenicity of an mRNA vectored Nipah virus vaccine candidate in pigs

**DOI:** 10.3389/fimmu.2024.1384417

**Published:** 2024-04-25

**Authors:** Miriam Pedrera, Rebecca K. McLean, Lobna Medfai, Nazia Thakur, Shawn Todd, Glenn Marsh, Dalan Bailey, Gaetano Donofrio, Hiromi Muramatsu, Norbert Pardi, Drew Weissman, Simon P. Graham

**Affiliations:** ^1^ The Pirbright Institute, Pirbright, United Kingdom; ^2^ Australian Centre for Disease Preparedness, Geelong, VIC, Australia; ^3^ Department of Medical-Veterinary Science, University of Parma, Parma, Italy; ^4^ Department of Medicine, Perelman School of Medicine, University of Pennsylvania, Philadelphia, PA, United States

**Keywords:** Nipah virus, vaccine, mRNA, pig, immunogenicity

## Abstract

Nipah virus (NiV) poses a significant threat to human and livestock populations across South and Southeast Asia. Vaccines are required to reduce the risk and impact of spillover infection events. Pigs can act as an intermediate amplifying host for NiV and, separately, provide a preclinical model for evaluating human vaccine candidate immunogenicity. The aim of this study was therefore to evaluate the immunogenicity of an mRNA vectored NiV vaccine candidate in pigs. Pigs were immunized twice with 100 μg nucleoside-modified mRNA vaccine encoding soluble G glycoprotein from the Malaysia strain of NiV, formulated in lipid nanoparticles. Potent antigen-binding and virus neutralizing antibodies were detected in serum following the booster immunization. Antibody responses effectively neutralized both the Malaysia and Bangladesh strains of NiV but showed limited neutralization of the related (about 80% amino acid sequence identity for G) Hendra virus. Antibodies were also capable of neutralizing NiV glycoprotein mediated cell-cell fusion. NiV G-specific T cell cytokine responses were also measurable following the booster immunization with evidence for induction of both CD4 and CD8 T cell responses. These data support the further evaluation of mRNA vectored NiV G as a vaccine for both pigs and humans.

## 1 Introduction

Nucleoside-modified mRNA has emerged as a versatile and highly effective vaccine platform to deliver antigens and elicit potent immune responses (1–3), while avoiding the anti-vector immunity associated with some live virus vaccines. mRNA immunization can elicit potent and durable neutralizing antibody responses capable of providing complete protection against viral pathogens (4, 5). In addition to showing promise in a range of pre-clinical models, mRNA vectored vaccines have played a major role in protecting human populations from the COVID-19 pandemic (3). Whilst mRNA vaccines will undoubtably play a prominent role as a human vaccine modality in the future, its potential to induce immunity in livestock species remains largely undetermined. Immunization of pigs with an mRNA vector encoding the rabies virus glycoprotein induced potent neutralizing antibodies following a booster immunization, which were comparable to those elicited by a licensed inactivated rabies virus vaccine (6). And a recent study, reported that an mRNA vector expressing the porcine epidemic diarrhea virus spike protein elicited neutralizing antibody titers comparable to those seen in pregnant sows immunized with an inactivated vaccine (7).

Nipah virus (NiV) poses a significant epidemic threat because of its broad host range and widespread distribution of *Pteropus* spp. bats which act as a natural reservoir. Humans may become infected indirectly from bats e.g., by consumption of contaminated raw date palm sap or through exposure to infected pigs or other livestock species. Pig-to-human transmission was responsible for the first and still most severe NiV outbreak in Malaysia and Singapore in 1998-99 (8). Despite the risk NiV poses, no vaccines are currently licensed for humans or pigs. Most experimental NiV vaccine approaches have used the envelope glycoprotein G formulated as either a recombinant protein subunit with adjuvant or expressed via viral vectors, with efficacy observed in a number of animal models, including pigs (9).

mRNA vectors have shown promise as NiV vaccine candidates. A single dose of mRNA expressing soluble G protein from the related Hendra virus protected hamsters from lethal NiV challenge (10) and an mRNA vector expressing a stabilized version of the NiV fusion protein covalently linked to three monomers of the NiV G protein induced potent antibody and T cell responses in mice (11). Building on these findings, the aim of this study was to evaluate the immunogenicity of nucleoside-modified mRNA expressing soluble NiV G protein in pigs.

## 2 Materials and methods

### 2.1 Generation and lipid nanoparticle formulation of mRNA encoding for the Nipah virus G protein

A synthetic nucleoside-modified mRNA encoding a soluble version of the G protein from the NiV Malaysia (NiV-M) strain (12) (mRNA-NiV sG) was formulated into lipid nanoparticles (LNPs). The mRNA was produced as previously described using T7 RNA polymerase (MEGAscript, Ambion) on a linearized plasmid encoding the codon-optimized antigen and a 101 nucleotide-long poly(A) tail (13). Instead of UTP, N-1-methylpseudouridine (m1Ψ) 5’-triphosphate (TriLink) was used to generate modified nucleoside-containing mRNA. During the *in vitro* transcription, co-transcriptional capping was performed using the trinucleotide cap1 analog, CleanCap (TriLink). The mRNA was purified by cellulose purification (14), as described, analyzed by gel electrophoresis and frozen at -20°C. The mRNA was encapsulated using an aqueous solution of mRNA at pH 4.0 and mixed with a solution of lipids dissolved in ethanol (15). The solution contains an ionizable cationic lipid/phosphatidylcholine/cholesterol/polyethylene glycol (PEG) lipid (proprietary of Acuitas, Vancouver, Canada) (50:10:38.5:1.5 mol/mol). RNA was mixed with the lipids at a ratio of ~0.05 (wt./wt.), LNP had a diameter of ~80 nm as measured by dynamic light scattering using a Zetasizer Nano ZS (Malvern Instruments Ltd, Malvern, UK) instrument, and stored at -80°C.

### 2.2 Recombinant NiV protein and peptides

Recombinant soluble NiV-M G protein (NiV sG) was expressed and purified as described previously (12). A pool of overlapping synthetic peptides (16mers offset by 4 amino acids) representing the NiV-M G protein were synthesized (Mimotopes, Melbourne, Australia) and used to stimulate T cells in IFN-γ ELISpot and intracellular cytokine staining (ICS) assays at a final concentration of 1 µg/mL per peptide (12).

### 2.3 Immunogenicity study

Six 8-10-week-old, female, Large White-Landrace-Hampshire cross-bred pigs were immunized by intramuscular inoculation of mRNA-NiV sG formulated in LNP (100 µg/dose in 1 mL). Animals received a homologous prime and booster immunization at 0- and 21-days post-vaccination (dpv). Animals were monitored daily (clinical signs and rectal temperature), blood samples were collected weekly at 0, 7, 14, 21, 28, 35, 42 dpv, and were euthanized on 42 dpv by pentobarbital overdose. The study was conducted in accordance with the UK Animals (Scientific Procedures) Act 1986 and with approval from the Animal Welfare and Ethical Review Body of the Animal and Plant Health Agency (APHA), Weybridge, UK.

### 2.4 Serum and peripheral blood mononuclear cell isolation

Serum and peripheral blood mononuclear cells (PBMC) were isolated as described previously (12). Briefly, SST tubes were centrifuged at 1,300 *x g*, for 10 minutes at room temperature (RT) and serum was collected and stored at -80°C. Heparinized blood was diluted in PBS and layered over 15 mL of Histopaque 1.077 (Merck Life Science) in Leucosep tubes (Thermo Fisher Scientific) and centrifugated with no break (800 *x g*, 15 minutes at RT). PBMCs were aspirated from the interface, the red blood cells lysed and PBMCs resuspended in RPMI-1640 medium (cRPMI) supplemented with 100 IU/mL penicillin, and 100 µg/mL streptomycin, 0.1% 2-mercaptoethanol and 10% heat inactivated (HI) fetal bovine serum (FBS) (all reagents from Thermo Fisher Scientific, Loughborough, UK), at the desired cell density and used immediately for the immunological assays or cryopreserved in cold 10% DMSO in HI FBS.

### 2.5 NiV-specific antibody responses

Detection of NiV G-specific antibodies was performed in sera as described previously (12). All serum samples were first tested at a 1:400 dilution. Serial dilutions of 21 and 42 dpv serum samples were then tested and end-point titers calculated as the reciprocal of the highest dilution at which the optical density (OD) value was greater than the cut-off value determined by a negative serum.

Detection of neutralizing antibodies in sera was performed by virus neutralization test (VNT) as previously described (16). All sera were tested for their ability to neutralize NiV-M. 42 dpv sera were additionally tested for their ability to neutralize NiV Bangladesh strain (NiV-B) and Hendra virus (HeV). Neutralization titers were expressed as the reciprocal of the serum dilution that completely blocked cytopathic effect (ND).

Detection of neutralizing antibodies was also carried out in all serum samples using NiV-M and NiV-B pseudoviruses as described (17). Pseudovirus neutralization titers were calculated as the inverse of the dilution which showed a 90% inhibition of luciferase values (IC_90_), compared to no serum controls.

Sera collected at 21 and 42 dpv were additionally assessed for neutralization of NiV glycoprotein-mediated cell–cell fusion using a quantitative fusion assay (17). The capacity of sera to inhibit NiV-M and NiV-B glycoprotein induced cell fusion was evaluated and calculated as the percentage of reduction of luciferase values compared to no sera control.

### 2.6 NiV-specific T cell cytokine responses

Porcine IFN-γ ELISpot assays were performed on peptide-stimulated PBMCs at 0, 7, 14, 21, 28, 35, 42 dpv to determine the frequency of NiV G-specific IFN-γ secreting cells, as described previously (12). The number of spots measured in unstimulated PBMC wells was subtracted from the spots measured in peptide stimulated PBMC wells and results presented as antigen-specific IFN-γ secreting cells per million cells.

Flow cytometric evaluation of IFN-γ and TNF-α expression induced by stimulation of PBMC with the NiV G peptide pool was also performed as described previously (12). Data were acquired using a MACSQuant Analyzer flow cytometer (Miltenyi Biotec) and analyzed with FlowJo software (BD Biosciences). The % cytokine expressing CD3^+^CD4^+^ cells and CD3^+^CD4^-^CD8α^high^ cells were determined (**Supplementary Figure 1**) and corrected by subtracting the background response obtained in unstimulated control cells for each pig.

### 2.7 Data analysis

GraphPad Prism 8.1.2 (GraphPad Software, San Diego, CA, USA) was used for graphical and statistical analysis of data sets. Statistical differences were analyzed using a repeat measure one-way or two-away ANOVA, with the Geisser-Greenhouse correction, and Tukey’s multiple comparison test, with individual variances computed for comparison between timepoints, or a paired t-test to compare antigen-specific cytokine and antibody responses at different time points post-vaccination as detailed in the results. Antibody titer data were log transformed before analysis. P-values < 0.05 were considered statistically significant.

## 3 Results

Clinical signs and temperatures were monitored daily for 7 days after prime and boost immunization. mRNA-NiV sG immunized pigs showed only a minimal increase in rectal temperature on 1 dpv (mean 39.2°C) (**Supplementary Figure 2**). No other local or systemic clinical signs were observed at any time post-vaccination (data not shown).

**Figure 1:**
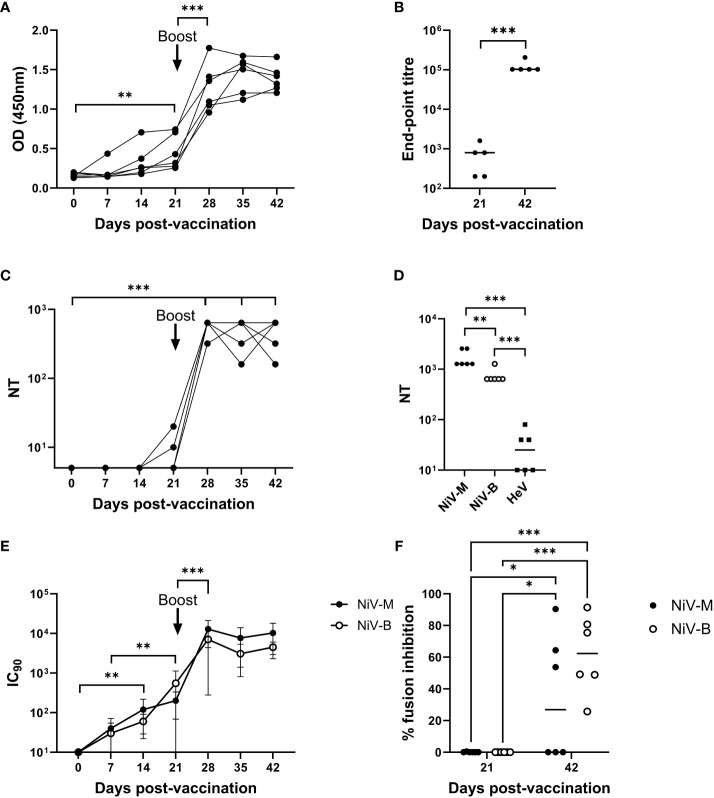
Antibody responses following immunization of pigs with mRNA vectored Nipah virus G protein. Pigs were immunized on 0 (prime) and 21 (boost) dpv. Recombinant NiV sG protein was used in ELISAs to assess antigen-specific antibody responses longitudinally **(A)** and end-point titers were determined in sera collected on 21 and 42 dpv **(B)**. Neutralizing antibody responses were assessed by classical VNT; longitudinal serum samples were assessed for neutralization of NiV-M **(C)** and day 42 sera tested for cross-neutralization of NiV-M, NiV-B, and HeV **(D)**. NiV neutralizing antibody titers were additionally assessed using NiV-M and NiV-B pseudoviruses **(E)** and presented as the reciprocal serum dilution to inhibit pseudovirus entry by 90% (IC_90_). Sera was assessed for inhibition of NiV-M and -B glycoprotein mediated cell–cell fusion **(F)**. Each data point represents individual pig sera with lines denoting the median. *p < 0.05; **p < 0.01; ***p < 0.001.

NiV G-specific antibody responses were assessed longitudinally in serum samples from the mRNA-NiV sG vaccinated pigs by indirect ELISA (**Figure 1A**). Antibody levels (OD values) significantly increased from 21 dpv (p<0.01) and were significantly boosted following the booster immunization (p<0.001), remaining elevated until the end of the study on 42 dpv. To quantify responses more accurately, NiV G-specific antibody endpoint titers were measured in sera at 21 and 42 dpv (**Figure 1B**). Comparison of serum antibody titers showed responses were significantly higher at 42 than at 21 dpv (p<0.001). NiV-M neutralizing antibody responses were evaluated longitudinally in sera using a classical VNT (**Figure 1C**). Low neutralizing antibody titers were observed in 2/6 pigs on 21 dpv and following the booster immunization neutralizing antibody titers rapidly rose in all six pigs (p<0.001) and were sustained until the end of the study (42 dpv). Sera from 42 dpv were additionally assessed for neutralization of NiV-M, NiV-B and HeV (**Figure 1D**). All pigs showed high neutralizing titers against both NiV strains, but only three animals displayed HeV neutralizing titers above the assay’s limit of detection. NiV-M and NiV-B pseudoviruses were neutralized by sera from all animals from 7 dpv, displaying a significant increase from 14 dpv with both pseudoviruses (p<0.001) (**Figure 1E**). Responses to both pseudoviruses were boosted following the second immunization, showing similar neutralizing titers from day 28 until the end of the study. Finally, the capacity of the serum samples from mRNA immunized pigs to neutralize NiV glycoprotein-mediated cell-cell fusion was assessed using cells which expressed NiV-M or NiV-B glycoproteins (**Figure 1F**). At 21 dpv, no inhibition was observed. However, sera collected at 42 dpv showed significant inhibition (p<0.05), with a surprisingly stronger effect being observed against NiV-B glycoprotein expressing cells.

**Figure 2:**
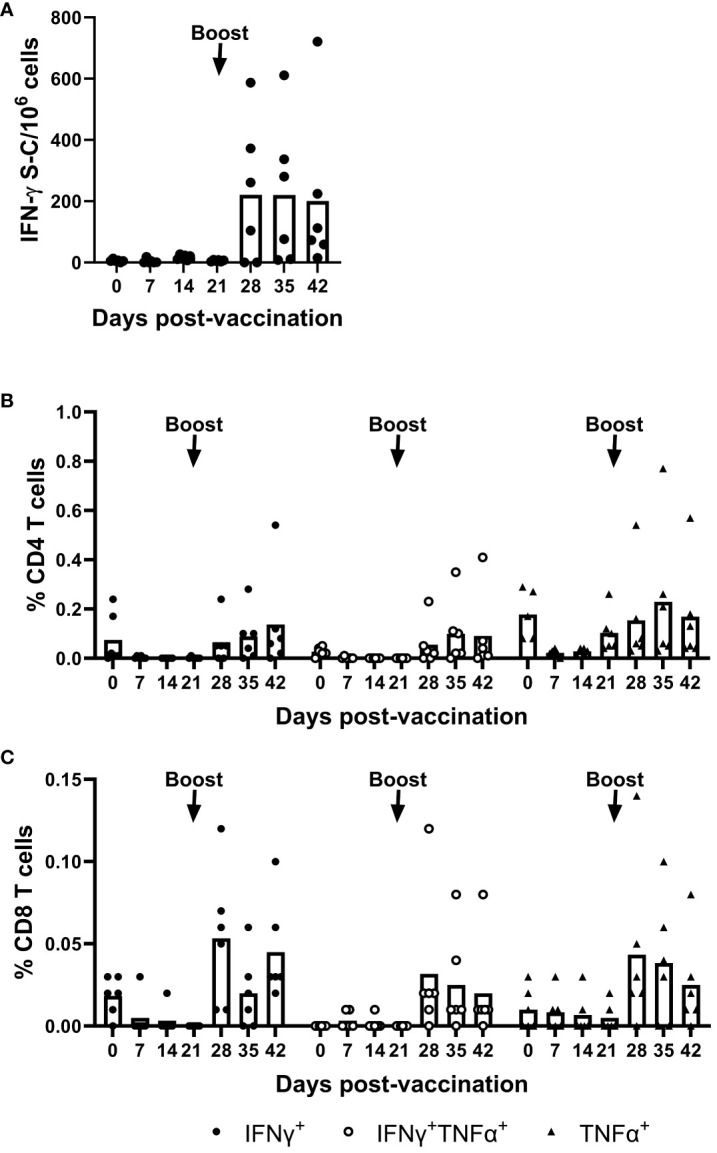
Evaluation of NiV antigen-specific T cell responses following immunization of pigs with mRNA vectored Nipah virus G protein. Pigs were immunized on 0 (prime) and 21 (boost) dpv. Responses of PBMC to stimulation with a NiV G peptide pool were monitored weekly by IFN-γ ELISpot assay and responding cells phenotyped by intracellular cytokine staining (ICS) assays. ELISpot data are presented as the unstimulated condition-corrected number of IFN-γ spot forming cells (S-C) per million PBMC **(A)** and ICS data shown as the unstimulated condition-corrected % cytokine expressing (IFN-γ and/or TNF-α) CD4^+^ **(B)** and CD8^+^ T cells **(C)**. Each data point represents individual pig PBMC responses with bars denoting the mean for each timepoint.

T cell responses induced by the mRNA-NiV sG vaccine were longitudinally assessed using an IFN-γ ELISpot assay following *ex vivo* stimulation of freshly isolated PBMCs with a pool of NiV G peptides (**Figure 2A**). Stimulation with the NiV G peptide pool induced a moderate IFN-γ response, but this response was only evident after the boost, from 28 dpv onwards. Responses post-boost showed significant inter-animal variability with 4/6 pigs showing clear peptide-specific responses, which meant responses did not reach statistical significance. Flow cytometry assays were also performed on PBMCs to phenotype the responding cells following NiV G peptide stimulation. IFN-γ and/or TNF-α expression by CD4 (CD3+CD8α-CD4+) and CD8 (CD3+CD8α+CD4-) T cells were assessed. A moderate increase in cytokine expressing CD4 T cells was observed in most pigs following the booster immunization (**Figure 2B**). A similar trend was observed for CD8 T cells post-boost, albeit with a lower frequency of responder cells (**Figure 2C**).

## 4 Discussion

NiV infection in humans causes severe respiratory and neurological disease, with a high fatality rate (40 – 75%) (18, 19). NiV infection in pigs causes a less severe disease, with no pathognomonic features. This makes early diagnosis difficult and allows the virus to be spread unnoticed. The eradication of NiV from the Malaysian pig herd required the culling of over 1 million animals. The development of a vaccine would be an important tool to prevent and reduce the impact of NiV outbreaks in pigs or people. To support this goal, we report here the evaluation of the immunogenicity of NiV G delivered to pigs by an LNP encapsulated nucleoside modified mRNA vector.

Immunization of the pigs with the mRNA vaccine candidate was safe with no localized or systemic adverse effects beyond a very modest rise in rectal temperatures 24 hours following the primary immunization. Longitudinal analysis of blood samples strongly suggested a requirement for a booster immunization to achieve high titer neutralizing antibodies and measurable CD4 and CD8 T cell responses. Whilst a single dose of mRNA has been shown to elicit potent immune responses in small animal models (20, 21), a booster immunization has typically been used in larger animals including the human COVID-19 vaccines (3). Although in the present study the inclusion of animals immunized with a single dose would have been necessary to confirm the impact of a booster dose, the data from prime-boost immunized pigs align with a previous evaluation of an mRNA vectored rabies vaccine in pigs, which showed a prominent increase in neutralizing titers post-boost (6). The NiV neutralizing titers observed post-boost were significantly greater than those elicited by an ALVAC vector expressing NiV G (12), a vaccine candidate that has been previously shown to confer pigs with a high level of protection against NiV (22). CD4 T cell and, to a lesser extent, CD8 T cell responses were detected following the booster immunization. The T cell responses were comparable to those observed in ALVAC NiV G immunized pigs but were inferior to the potent responses elicited by a bovine herpes virus 4 vector (12). This may be significant since data from a study evaluating the protective efficacy of a HeV sG protein-based vaccine suggested that both antibody and cell-mediated immunity is necessary to protect pigs against henipaviruses (23).

We conclude that the responses observed following prime-boost immunization with the mRNA NiV G vaccine candidate merit its continued evaluation including its ability to confer protection against Nipah virus infection and disease in pigs and other relevant preclinical models.

## Data availability statement

The original contributions presented in the study are included in the article/**Supplementary Material**. Further inquiries can be directed to the corresponding authors.

## Ethics statement

The animal study was approved by Animal Welfare and Ethical Review Body of the Animal and Plant Health Agency (APHA), Weybridge, UK. The study was conducted in accordance with the local legislation and institutional requirements.

## Author contributions

MP: Writing – original draft, Formal analysis, Investigation, Supervision, Visualization. RM: Writing – review & editing, Formal analysis, Investigation. LM: Writing – review & editing, Investigation. NT: Writing – review & editing, Formal analysis, Investigation. ST: Writing – review & editing, Investigation. GM: Writing – review & editing, Supervision. DB: Writing – review & editing, Supervision. GD: Writing – review & editing, Funding acquisition. HM: Writing – review & editing, Investigation. NP: Writing – review & editing, Investigation. DW: Writing – review & editing, Conceptualization, Supervision. SG: Writing – original draft, Conceptualization, Funding acquisition, Project administration.

## References

[1] Pardi N Hogan MJ Porter FW Weissman D. mRNA vaccines - a new era in vaccinology. Nat Rev Drug Discov. (2018) 17:261–79. doi: 10.1038/nrd.2017.243 PMC5906799 29326426

[2] Alameh MG Weissman D Pardi N. Messenger RNA-based vaccines against infectious diseases. Curr Top Microbiol Immunol. (2022) 440:111–45. doi: 10.1007/82_2020_202 32300916

[3] Hogan MJ Pardi N. mRNA vaccines in the COVID-19 pandemic and beyond. Annu Rev Med. (2022) 73:17–39. doi: 10.1146/annurev-med-042420-112725 34669432

[4] Pardi N Hogan MJ Pelc RS Muramatsu H Andersen H DeMaso CR. Zika virus protection by a single low-dose nucleoside-modified mRNA vaccination. Nature. (2017) 543:248–51. doi: 10.1038/nature21428 PMC5344708 28151488

[5] Pardi N Carreño JM O'Dell G Tan J Bajusz C Muramatsu H. Development of a pentavalent broadly protective nucleoside-modified mRNA vaccine against influenza B viruses. Nat Commun. (2022) 13:4677. doi: 10.1038/s41467-022-32149-8 35945226 PMC9362976

[6] Schnee M Vogel AB Voss D Petsch B Baumhof P Kramps T. An mRNA Vaccine Encoding Rabies Virus Glycoprotein Induces Protection against Lethal Infection in Mice and Correlates of Protection in Adult and Newborn Pigs. PLoS Negl Trop Dis. (2016) 10:e0004746. doi: 10.1371/journal.pntd.0004746 27336830 PMC4918980

[7] Yang L Wang J Xu M Wang H Zhang X Liu W. [Preparation and immunogenicity evaluation of mRNA vaccine against porcine epidemic diarrhea]. Sheng Wu Gong Cheng Xue Bao. (2023) 39:2624–33. doi: 10.13345/j.cjb.220853 37584119

[8] McLean RK Graham SP. Vaccine development for Nipah virus infection in pigs. Front Vet Sci. (2019) 6:16. doi: 10.3389/fvets.2019.00016 30778392 PMC6369168

[9] Satterfield BA Mire CE Geisbert TW. Overview of experimental vaccines and antiviral therapeutics for Henipavirus infection. In: Freiberg AN Rockx B, editors. Nipah Virus: Methods and Protocols. Springer US, New York, NY (2023). p. 1–22. 10.1007/978-1-0716-3283-3_1 37610570

[10] Lo MK Spengler JR Welch SR Harmon JR Coleman-McCray JD Scholte FEM. Evaluation of a single-dose nucleoside-modified messenger RNA vaccine encoding Hendra virus-soluble glycoprotein against lethal Nipah virus challenge in Syrian hamsters. J Infect Dis. (2020) 221:S493–s498. doi: 10.1093/infdis/jiz553 31751453 PMC7368163

[11] Loomis RJ DiPiazza AT Falcone S Ruckwardt TJ Morabito KM Abiona OM. Chimeric fusion (F) and attachment (G) glycoprotein antigen delivery by mRNA as a candidate Nipah vaccine. Front Immunol. (2021) 12:772864. doi: 10.3389/fimmu.2021.772864 34956199 PMC8692728

[12] Pedrera M Macchi F McLean RK Franceschi V Thakur N Russo L. Bovine Herpesvirus-4-vectored delivery of Nipah virus glycoproteins enhances T cell immunogenicity in pigs. Vaccines (Basel). (2020) 8(1):115. doi: 10.3390/vaccines8010115 32131403 PMC7157636

[13] Kunkeaw N Nguitragool W Takashima E Kangwanrangsan N Muramatsu H Tachibana M. A Pvs25 mRNA vaccine induces complete and durable transmission-blocking immunity to Plasmodium vivax. NPJ Vaccines. (2023) 8:187. doi: 10.1038/s41541-023-00786-9 38092803 PMC10719277

[14] Baiersdörfer M Boros G Muramatsu H Mahiny A Vlatkovic I Sahin U. A Facile Method for the Removal of dsRNA Contaminant from *In Vitro*-Transcribed mRNA. Mol Ther Nucleic Acids. (2019) 15:26–35. doi: 10.1016/j.omtn.2019.02.018 30933724 PMC6444222

[15] Maier MA Jayaraman M Matsuda S Liu J Barros S Querbes W. Biodegradable lipids enabling rapidly eliminated lipid nanoparticles for systemic delivery of RNAi therapeutics. Mol Ther. (2013) 21:1570–8. doi: 10.1038/mt.2013.124 PMC3734658 23799535

[16] Crameri G Wang L-F Morrissy C White J Eaton BT. A rapid immune plaque assay for the detection of Hendra and Nipah viruses and anti-virus antibodies. J Virological Methods. (2002) 99:41–51. doi: 10.1016/S0166-0934(01)00377-9 11684302

[17] Thakur N Conceicao C Isaacs A Human S Modhiran N McLean RK. Micro-fusion inhibition tests: quantifying antibody neutralization of virus-mediated cell–cell fusion. J Gen Virol. (2021) 102(1):001506. doi: 10.1099/jgv.0.001506 PMC8116787 33054904

[18] Chua KB Bellini WJ Rota PA Harcourt BH Tamin A Lam SK. Nipah virus: A recently emergent deadly paramyxovirus. Science. (2000) 288:1432–5. doi: 10.1126/science.288.5470.1432 10827955

[19] Donaldson H Lucey D. Enhancing preparation for large Nipah outbreaks beyond Bangladesh: Preventing a tragedy like Ebola in West Africa. Int J Infect Dis. (2018) 72:69–72. doi: 10.1016/j.ijid.2018.05.015 29879523 PMC7110759

[20] Pardi N Parkhouse K Kirkpatrick E McMahon M Zost SJ Mui BL. Nucleoside-modified mRNA immunization elicits influenza virus hemagglutinin stalk-specific antibodies. Nat Commun. (2018) 9:3361. doi: 10.1038/s41467-018-05482-0 30135514 PMC6105651

[21] McMahon M O'Dell G Tan J Sárközy A Vadovics M Carreño JM. Assessment of a quadrivalent nucleoside-modified mRNA vaccine that protects against group 2 influenza viruses. Proc Natl Acad Sci USA. (2022) 119:e2206333119. doi: 10.1073/pnas.2206333119 36322769 PMC9659346

[22] Weingartl HM Berhane Y Caswell JL Loosmore S Audonnet JC Roth JA. Recombinant nipah virus vaccines protect pigs against challenge. J Virol. (2006) 80:7929–38. doi: 10.1128/JVI.00263-06 PMC1563797 16873250

[23] Pickering BS Hardham JM Smith G Weingartl ET Dominowski PJ Foss DL. Protection against henipaviruses in swine requires both, cell-mediated and humoral immune response. Vaccine. (2016) 34:4777–86. doi: 10.1016/j.vaccine.2016.08.028 PMC6161494 27544586

