# Supplementary material for: Evaluation of the immunogenicity of an mRNA vectored Nipah virus vaccine candidate in pigs
Source: Front Immunol. 2024 Apr 25;15:1384417. doi: 10.3389/fimmu.2024.1384417 (PMC11079202; doi:10.3389/fimmu.2024.1384417)
Supplement: Supplementary file 1 [file DataSheet_1.pdf]

*Supplementary Material*

# Evaluation of the immunogenicity of an mRNA vectored Nipah virus vaccine candidate in pigs

**Miriam Pedrera, Rebecca K. McLean, Lobna Medfai, Nazia Thakur, Shawn Todd, Glenn A. Marsh, Dalan Bailey, Gaetano Donofrio, Hiromi Muramatsu, Norbert Pardi, Drew Weissman, Simon P. Graham**

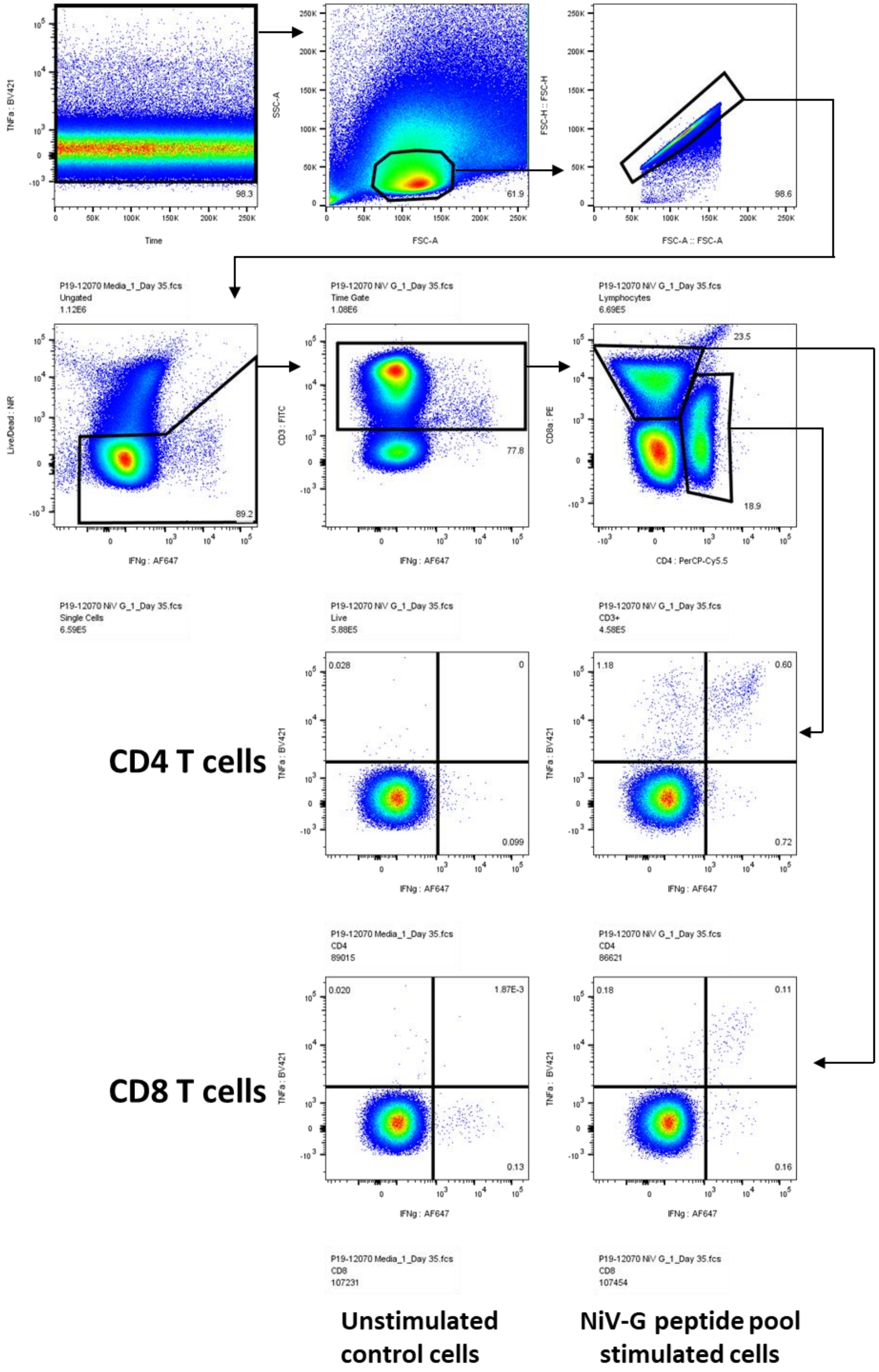


**Supplementary Figure 1.** Illustration of gating strategy to assess cytokine responses of unstimulated and NiV G peptide stimulated porcine PBMC.

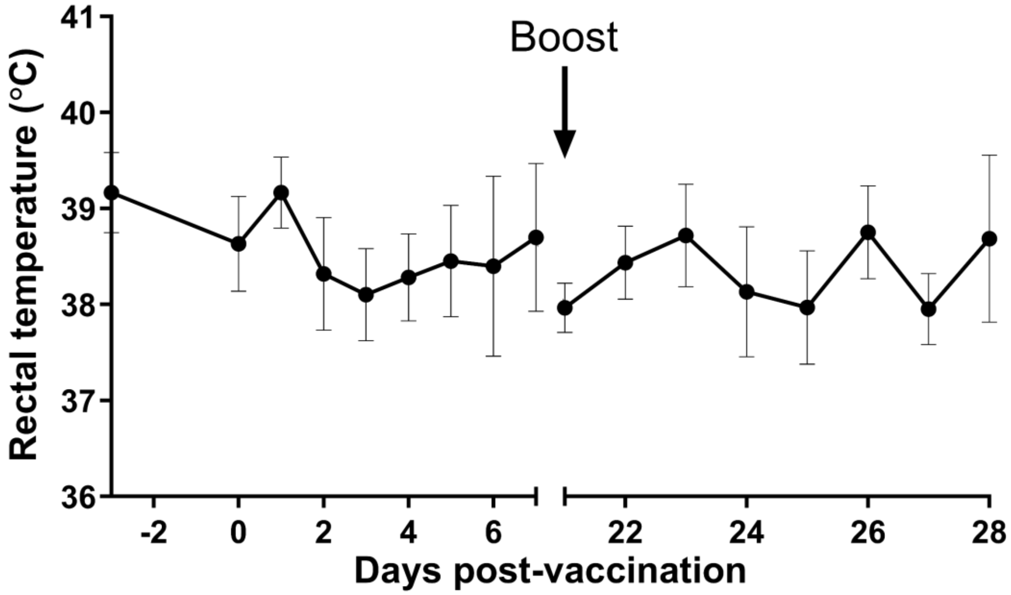


**Supplementary Figure 2.** Evaluation of the reactogenicity of an mRNA vectored Nipah virus vaccine candidate in pigs. Pigs (n=6) were primed with LNP formulated mRNA-NiV G at day 0 and boosted at day 21. Rectal temperatures were measured for 7 days following prime and boost immunization. Mean rectal temperatures ± SD are shown.
